# Hexakis(dimethyl­ammonium) di-μ_6_-oxido-tetra-μ_3_-oxido-tetra­deca-μ_2_-oxido-octa­oxidodeca­vanadate(V) monohydrate

**DOI:** 10.1107/S1600536810016442

**Published:** 2010-05-08

**Authors:** Sulian Wang, Liping Lu, Sisi Feng, Miaoli Zhu

**Affiliations:** a Institute of Molecular Science, Key Laboratory of Chemical Biology and Molecular Engineering of the Education Ministry, Shanxi University, Taiyuan, Shanxi 030006, People’s Republic of China

## Abstract

In the title compound, (C_2_H_8_N)_6_[V_10_O_28_]·H_2_O, the [V_10_O_28_]^6−^ polymetalate anion has crystallographic mirror symmetry with six V atoms and 12 O atoms lying on the mirror plane. Each of the V^V^ atoms adopts a distorted octa­hedral geometry. Eight terminal O atoms are bonded to V^V^ atoms with double bonds and the others act as bridging atoms. In the crystal structure, a network of N—H⋯O and O—H⋯O hydrogen bonds helps to establish the packing.

## Related literature

For the biological activity of oxovanadates and vanadium complexes, see: Pacigová *et al.* (2007 ▶); Yuan, Lu, Gao *et al.* (2009 ▶). For a related structure, see: Yuan, Lu, Zhu *et al.* (2009 ▶).
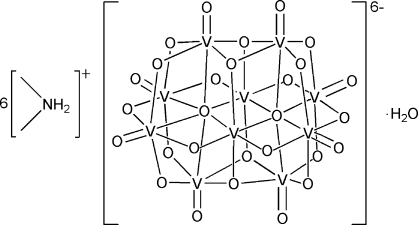


## Experimental

### 

#### Crystal data

(C_2_H_8_N)_6_[V_10_O_28_]·H_2_O
*M* *_r_* = 1251.98
Orthorhombic, 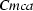

*a* = 13.6149 (18) Å
*b* = 18.629 (3) Å
*c* = 30.235 (2) Å
*V* = 7668.5 (16) Å^3^
*Z* = 8
Mo *K*α radiation
μ = 2.42 mm^−1^
*T* = 203 K
0.35 × 0.11 × 0.05 mm

#### Data collection

Bruker SMART 1K CCD diffractometer
Absorption correction: multi-scan (*SADABS*; Sheldrick, 1996 ▶) *T*_min_ = 0.484, *T*_max_ = 0.888
18064 measured reflections
3464 independent reflections
2951 reflections with *I* > 2σ(*I*)
*R*_int_ = 0.078

#### Refinement

*R*[*F* ^2^ > 2σ(*F* ^2^)] = 0.096
*wR*(*F* ^2^) = 0.177
*S* = 1.26
3464 reflections
296 parameters
6 restraints
H-atom parameters constrained
Δρ_max_ = 1.70 e Å^−3^
Δρ_min_ = −0.83 e Å^−3^

### 

Data collection: *SMART* (Bruker, 2007 ▶); cell refinement: *SAINT* (Bruker, 2007 ▶); data reduction: *SAINT* (Bruker, 2007 ▶); program(s) used to solve structure: *SHELXS97* (Sheldrick, 2008 ▶); program(s) used to refine structure: *SHELXL97* (Sheldrick, 2008 ▶); molecular graphics: *SHELXTL* (Sheldrick, 2008 ▶); software used to prepare material for publication: *SHELXTL* (Sheldrick, 2008 ▶).

## Supplementary Material

Crystal structure: contains datablocks I, global. DOI: 10.1107/S1600536810016442/hy2304sup1.cif

Structure factors: contains datablocks I. DOI: 10.1107/S1600536810016442/hy2304Isup2.hkl

Additional supplementary materials:  crystallographic information; 3D view; checkCIF report

## Figures and Tables

**Table 1:** Hydrogen-bond geometry (Å, °)

*D*—H⋯*A*	*D*—H	H⋯*A*	*D*⋯*A*	*D*—H⋯*A*
O21—H21*B*⋯O10^i^	0.85	2.30	2.950 (19)	133
O21—H21*A*⋯O16	0.85	2.26	3.101 (18)	171
N4—H4*D*⋯O5^ii^	0.91	1.77	2.673 (8)	170
N4—H4*E*⋯O5	0.91	1.77	2.673 (8)	170
N2—H2*D*⋯O6	0.91	1.88	2.759 (10)	162
N2—H2*E*⋯O6^iii^	0.91	2.22	2.986 (10)	141
N2—H2*E*⋯O7^iii^	0.91	2.05	2.812 (9)	140
N1—H1*A*⋯O8^iv^	0.91	1.86	2.732 (10)	161
N1—H1*B*⋯O3	0.91	1.79	2.674 (10)	164

Symmetry codes: (i) 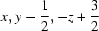; (ii) 
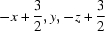
; (iii) 
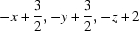
; (iv) 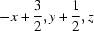.

**Fig. 1.**
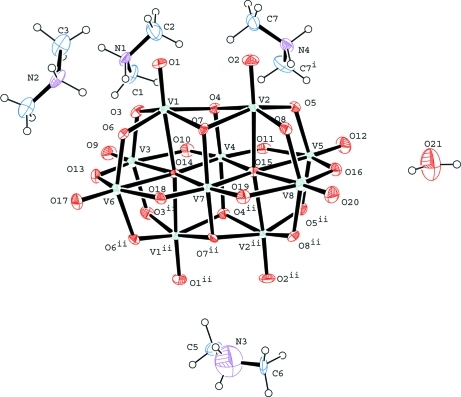
The structure of the title compound. Displacement ellipsoids are drawn at the 30% probability level. [Symmetry codes: (i) 3/2-x, y, 3/2-z; (ii) 1-x, y, z.]

**Fig. 2.**
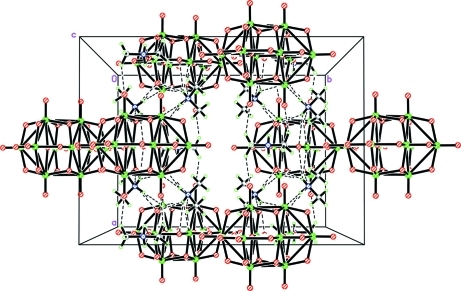
The crystal packing in the title compound. Hydrogen bonds are indicated by dashed lines. [Red, O; green, V; blue, N; gray, C and green open circle, H.]

## supplementary crystallographic information

### Comment

Oxovanadates(V) and peroxovanadium compounds are of great interest in biochemistry and medicine because of their diverse biological activites (Pacigová *et al.*, 2007). Of them, decavanadates have shown high affinity for selected kinases and phosphorylase and have been used to facilitate crystallization of proteins. Vanadium complexes can inhibit effectively activity of protein tyrosine phosphatase (Yuan, Lu, Gao *et al.*, 2009). In our previous work, (C_5_H_7_N_2_)_6_[V_10_O_28_].2H_2_O was reported (Yuan, Lu, Zhu *et al.*, 2009). Herein, we report the structure of the title compound.

The title compound consists of a [V_10_O_28_]^6-^ polyanion, six dimethylaminium cations and one water molecule (Fig. 1). The polyanion is constructed by ten edge-sharing VO_6_ octahedra. Six V atoms and twelve O atoms lie on the mirror plane at x = 1/2. Different coordination O atoms existing in the anion result in differnet V—O bond distances. The V—O(terminal) double bond distances range from 1.551 (10) to 1.576 (6) Å, shorter than those in (C_5_H_7_N_2_)_6_[V_10_O_28_].2H_2_O (Yuan, Lu, Zhu *et al.*, 2009). The V—O(µ_3_) single bond distances range from 1.638 (10) to 2.066 (10) Å. The V—O (µ_2_) single bond distances range from 1.897 (6) to 1.991 (6)Å. The V—O (µ_6_) single bond distances are more longer [2.137 (8) to 2.275 (9)Å].

A three-dimensional supramolecular hydrogen-bonding network is observed in the crystal structure and details are given in Table 1 and Fig. 2.

### Experimental

A mixture containing 1.5 mmol each of VO(acac)_2_ (acac = acetylacetone), 1,10-phenanthroline and 2-(2-hydroxylphenyl)benzimidazole in methanol (24 ml) was refluxed for 30 min. Light green precipitate was filtrated and collected. The solid was dissolved in dimethylformamide. The solvent was slowly evaporated for one month and yellow crystals of the title compound were obtained.

### Refinement

The highest residual electron density was found 0.48 and 0.72 Å from H3D and N3 and the deepest hole 1.02 Å from O15. H atoms except those of water were included in calculated positions and treated as riding atoms, with C—H = 0.97 and N—H = 0.91 Å and with *U*_iso_(H) = 1.2(1.5 for methyl)*U*_eq_(C,N). H atoms attached to water molecule were located in a difference Fourier map and refined as riding, with O—H = 0.85 Å and *U*_iso_(H) = *U*_eq_(O).

### Crystal data

**Table:**

(C_2_H_8_N)_6_[V_10_O_28_]·H_2_O	*F*(000) = 5008
*M**_r_* = 1251.98	*D*_x_ = 2.169 Mg m^−^^3^
Orthorhombic, *C**m**c**a*	Mo *K*α radiation, λ = 0.71073 Å
Hall symbol: -C 2bc 2	Cell parameters from 1649 reflections
*a* = 13.6149 (18) Å	θ = 2.3–22.0°
*b* = 18.629 (3) Å	µ = 2.42 mm^−^^1^
*c* = 30.235 (2) Å	*T* = 203 K
*V* = 7668.5 (16) Å^3^	Needle, yellow
*Z* = 8	0.35 × 0.11 × 0.05 mm

### Data collection

**Table:**

Bruker SMART 1K CCD diffractometer	3464 independent reflections
Radiation source: fine-focus sealed tube	2951 reflections with *I* > 2σ(*I*)
graphite	*R*_int_ = 0.078
φ and ω scans	θ_max_ = 25.0°, θ_min_ = 2.0°
Absorption correction: multi-scan (*SADABS*; Sheldrick, 1996)	*h* = −15→16
*T*_min_ = 0.484, *T*_max_ = 0.888	*k* = −22→22
18064 measured reflections	*l* = −35→28

### Refinement

**Table:**

Refinement on *F*^2^	Primary atom site location: structure-invariant direct methods
Least-squares matrix: full	Secondary atom site location: difference Fourier map
*R*[*F*^2^ > 2σ(*F*^2^)] = 0.096	Hydrogen site location: inferred from neighbouring sites
*wR*(*F*^2^) = 0.177	H-atom parameters constrained
*S* = 1.26	*w* = 1/[σ^2^(*F*_o_^2^) + (0.0331*P*)^2^ + 151.3848*P*] where *P* = (*F*_o_^2^ + 2*F*_c_^2^)/3
3464 reflections	(Δ/σ)_max_ = 0.001
296 parameters	Δρ_max_ = 1.70 e Å^−^^3^
6 restraints	Δρ_min_ = −0.82 e Å^−^^3^

### Special details

**Table:**

Refinement. The structure was phased by direct methods. The space group choice was confirmed by successful convergence of the full-matrix least-squares refinement on *F*^2^. The Ueq of N3 is large. It is resulted from the severe systematic disorder of N3 atom, which is located at crystallographic mirror symmetry. ISOR instruction was employed to have ellipsoids of site N3 be restraint to more appropriate values. So, 6 restraints were used for anisotropic refinement.

### Fractional atomic coordinates and isotropic or equivalent isotropic displacement parameters (Å^2^)

**Table:**

	*x*	*y*	*z*	*U*_iso_*/*U*_eq_	Occ. (<1)
V1	0.66109 (11)	0.75463 (8)	0.90694 (5)	0.0247 (4)	
V2	0.66020 (10)	0.61321 (8)	0.86058 (5)	0.0238 (4)	
V3	0.5000	0.86507 (12)	0.89222 (8)	0.0312 (6)	
V4	0.5000	0.72904 (12)	0.83754 (7)	0.0236 (5)	
V5	0.5000	0.58253 (13)	0.79179 (7)	0.0263 (5)	
V6	0.5000	0.78222 (13)	0.97603 (8)	0.0290 (6)	
V7	0.5000	0.63836 (11)	0.92977 (7)	0.0197 (5)	
V8	0.5000	0.50136 (12)	0.87744 (8)	0.0295 (6)	
O1	0.7763 (4)	0.7541 (4)	0.9081 (2)	0.0376 (17)	
O2	0.7759 (4)	0.6129 (4)	0.8602 (2)	0.0385 (17)	
O3	0.6330 (5)	0.8441 (3)	0.8900 (2)	0.0338 (17)	
O4	0.6366 (4)	0.7163 (3)	0.8497 (2)	0.0245 (14)	
O5	0.6308 (4)	0.5943 (3)	0.8034 (2)	0.0266 (14)	
O6	0.6325 (4)	0.7704 (3)	0.9642 (2)	0.0269 (15)	
O7	0.6361 (4)	0.6511 (3)	0.91878 (19)	0.0218 (13)	
O8	0.6329 (4)	0.5241 (3)	0.8785 (2)	0.0273 (14)	
O9	0.5000	0.9456 (5)	0.8775 (4)	0.047 (3)	
O10	0.5000	0.8170 (5)	0.8307 (3)	0.035 (2)	
O11	0.5000	0.6906 (5)	0.7888 (3)	0.031 (2)	
O12	0.5000	0.5657 (6)	0.7412 (3)	0.049 (3)	
O13	0.5000	0.8687 (5)	0.9508 (3)	0.034 (2)	
O14	0.5000	0.7460 (4)	0.9052 (3)	0.0171 (17)	
O15	0.5000	0.6211 (4)	0.8632 (3)	0.0229 (19)	
O16	0.5000	0.4958 (5)	0.8191 (3)	0.031 (2)	
O17	0.5000	0.8004 (5)	1.0263 (3)	0.041 (3)	
O18	0.5000	0.6771 (5)	0.9790 (3)	0.027 (2)	
O19	0.5000	0.5509 (5)	0.9370 (3)	0.030 (2)	
O20	0.5000	0.4213 (5)	0.8915 (4)	0.048 (3)	
O21	0.5000	0.3555 (9)	0.7640 (5)	0.119 (6)	
H21A	0.5000	0.3911	0.7816	0.119*	
H21B	0.5000	0.3717	0.7377	0.119*	
N1	0.7294 (6)	0.9384 (4)	0.8394 (3)	0.037 (2)	
H1A	0.7635	0.9719	0.8551	0.044*	
H1B	0.6913	0.9134	0.8588	0.044*	
C1	0.6667 (9)	0.9744 (7)	0.8076 (4)	0.058 (3)	
H1C	0.7061	1.0065	0.7895	0.087*	
H1D	0.6170	1.0019	0.8231	0.087*	
H1E	0.6353	0.9390	0.7888	0.087*	
C2	0.7972 (10)	0.8902 (7)	0.8190 (5)	0.066 (4)	
H2A	0.7615	0.8509	0.8054	0.099*	
H2B	0.8419	0.8714	0.8411	0.099*	
H2C	0.8343	0.9156	0.7965	0.099*	
N2	0.7745 (6)	0.8610 (4)	0.9977 (3)	0.040 (2)	
H2D	0.7384	0.8266	0.9839	0.048*	
H2E	0.8145	0.8386	1.0175	0.048*	
C3	0.8341 (10)	0.8966 (7)	0.9655 (4)	0.063 (4)	
H3A	0.7924	0.9230	0.9452	0.094*	
H3B	0.8720	0.8613	0.9492	0.094*	
H3C	0.8784	0.9296	0.9803	0.094*	
C4	0.7104 (10)	0.9067 (7)	1.0209 (4)	0.061 (4)	
H4A	0.7484	0.9405	1.0383	0.092*	
H4B	0.6691	0.8784	1.0403	0.092*	
H4C	0.6697	0.9327	1.0000	0.092*	
N3	0.0000	0.6868 (16)	0.8841 (10)	0.177 (12)	
H3D	−0.0530	0.6904	0.9023	0.177*	0.50
H3E	0.0530	0.6904	0.9023	0.177*	0.50
C5	0.0000	0.7485 (9)	0.8640 (6)	0.052 (4)	
H5A	0.0665	0.7669	0.8626	0.052*	0.50
H5B	−0.0412	0.7821	0.8800	0.052*	0.50
H5C	−0.0254	0.7422	0.8343	0.052*	0.50
C6	0.0000	0.6194 (9)	0.8737 (6)	0.060 (5)	
H6A	−0.0588	0.5967	0.8852	0.060*	0.50
H6B	0.0576	0.5963	0.8861	0.060*	0.50
H6C	0.0000	0.6147	0.8417	0.060*	
N4	0.7500	0.6685 (6)	0.7500	0.040 (3)	
H4D	0.7849	0.6396	0.7315	0.047*	0.50
H4E	0.7151	0.6396	0.7685	0.047*	0.50
C7	0.8174 (10)	0.7099 (7)	0.7757 (4)	0.066 (4)	
H7A	0.8489	0.7453	0.7569	0.099*	
H7B	0.8668	0.6785	0.7883	0.099*	
H7C	0.7822	0.7341	0.7993	0.099*	

### Atomic displacement parameters (Å^2^)

**Table:**

	*U*^11^	*U*^22^	*U*^33^	*U*^12^	*U*^13^	*U*^23^
V1	0.0161 (8)	0.0257 (8)	0.0322 (9)	−0.0038 (7)	−0.0006 (7)	−0.0023 (7)
V2	0.0152 (7)	0.0285 (8)	0.0277 (9)	0.0032 (6)	−0.0005 (7)	−0.0034 (7)
V3	0.0291 (13)	0.0183 (12)	0.0462 (16)	0.000	0.000	0.0019 (11)
V4	0.0233 (12)	0.0268 (12)	0.0207 (12)	0.000	0.000	0.0065 (10)
V5	0.0235 (12)	0.0358 (13)	0.0198 (12)	0.000	0.000	−0.0055 (10)
V6	0.0265 (12)	0.0349 (13)	0.0255 (13)	0.000	0.000	−0.0098 (11)
V7	0.0175 (10)	0.0250 (11)	0.0167 (11)	0.000	0.000	0.0037 (9)
V8	0.0340 (13)	0.0195 (11)	0.0351 (14)	0.000	0.000	0.0020 (10)
O1	0.018 (3)	0.044 (4)	0.051 (5)	−0.008 (3)	0.001 (3)	−0.006 (4)
O2	0.017 (3)	0.057 (5)	0.041 (4)	0.006 (3)	−0.003 (3)	−0.002 (4)
O3	0.031 (4)	0.027 (3)	0.044 (4)	−0.014 (3)	−0.001 (3)	0.004 (3)
O4	0.020 (3)	0.026 (3)	0.027 (3)	0.000 (3)	−0.001 (3)	0.003 (3)
O5	0.025 (3)	0.025 (3)	0.030 (4)	−0.003 (3)	0.007 (3)	−0.006 (3)
O6	0.020 (3)	0.033 (4)	0.028 (3)	−0.006 (3)	−0.003 (3)	−0.009 (3)
O7	0.020 (3)	0.024 (3)	0.021 (3)	0.000 (3)	−0.008 (3)	−0.004 (3)
O8	0.023 (3)	0.023 (3)	0.035 (4)	0.011 (3)	−0.002 (3)	0.000 (3)
O9	0.042 (6)	0.035 (6)	0.063 (8)	0.000	0.000	0.001 (5)
O10	0.037 (6)	0.032 (5)	0.035 (6)	0.000	0.000	0.009 (4)
O11	0.021 (5)	0.035 (5)	0.035 (6)	0.000	0.000	0.010 (4)
O12	0.052 (7)	0.071 (8)	0.023 (6)	0.000	0.000	−0.014 (5)
O13	0.036 (5)	0.030 (5)	0.038 (6)	0.000	0.000	−0.012 (5)
O14	0.016 (4)	0.012 (4)	0.023 (5)	0.000	0.000	0.004 (4)
O15	0.019 (4)	0.028 (5)	0.022 (5)	0.000	0.000	0.006 (4)
O16	0.035 (5)	0.034 (5)	0.024 (5)	0.000	0.000	−0.003 (4)
O17	0.039 (6)	0.050 (6)	0.034 (6)	0.000	0.000	−0.018 (5)
O18	0.023 (5)	0.041 (5)	0.017 (5)	0.000	0.000	−0.004 (4)
O19	0.033 (5)	0.025 (5)	0.033 (6)	0.000	0.000	0.008 (4)
O20	0.053 (7)	0.029 (6)	0.062 (8)	0.000	0.000	−0.004 (5)
O21	0.172 (18)	0.101 (13)	0.084 (13)	0.000	0.000	−0.009 (10)
N1	0.045 (5)	0.030 (4)	0.036 (5)	−0.017 (4)	−0.001 (4)	−0.004 (4)
C1	0.066 (8)	0.060 (8)	0.048 (7)	−0.017 (7)	−0.014 (7)	−0.009 (6)
C2	0.069 (9)	0.055 (8)	0.074 (10)	0.017 (7)	0.000 (7)	−0.015 (7)
N2	0.047 (5)	0.035 (5)	0.039 (5)	−0.013 (4)	−0.029 (5)	0.011 (4)
C3	0.080 (10)	0.059 (8)	0.049 (8)	−0.018 (7)	−0.014 (7)	0.017 (6)
C4	0.064 (9)	0.052 (7)	0.068 (9)	−0.013 (7)	−0.022 (7)	−0.008 (7)
N3	0.207 (15)	0.163 (14)	0.160 (15)	0.000	0.000	−0.003 (10)
C5	0.036 (9)	0.045 (10)	0.075 (13)	0.000	0.000	0.003 (10)
C6	0.057 (11)	0.045 (10)	0.078 (14)	0.000	0.000	−0.042 (10)
N4	0.044 (7)	0.035 (7)	0.040 (7)	0.000	0.019 (6)	0.000
C7	0.068 (9)	0.065 (8)	0.064 (9)	−0.013 (7)	0.032 (7)	−0.031 (7)

### Geometric parameters (Å, °)

**Table:**

V1—O1	1.569 (6)	V8—O15	2.272 (9)
V1—O3	1.784 (7)	O21—H21A	0.8500
V1—O6	1.799 (6)	O21—H21B	0.8500
V1—O4	1.902 (6)	N1—C2	1.428 (13)
V1—O7	1.991 (6)	N1—C1	1.452 (14)
V1—O14	2.200 (2)	N1—H1A	0.9100
V2—O2	1.576 (6)	N1—H1B	0.9100
V2—O8	1.785 (6)	C1—H1C	0.9700
V2—O5	1.810 (6)	C1—H1D	0.9700
V2—O7	1.924 (6)	C1—H1E	0.9700
V2—O4	1.975 (6)	C2—H2A	0.9700
V2—O15	2.188 (2)	C2—H2B	0.9700
V3—O9	1.565 (10)	C2—H2C	0.9700
V3—O13	1.771 (10)	N2—C4	1.406 (14)
V3—O3	1.854 (7)	N2—C3	1.431 (14)
V3—O10	2.066 (10)	N2—H2D	0.9100
V3—O14	2.254 (8)	N2—H2E	0.9100
V4—O11	1.638 (10)	C3—H3A	0.9700
V4—O10	1.651 (9)	C3—H3B	0.9700
V4—O4	1.910 (6)	C3—H3C	0.9700
V4—O14	2.071 (8)	C4—H4A	0.9700
V4—O15	2.155 (8)	C4—H4B	0.9700
V5—O12	1.561 (10)	C4—H4C	0.9700
V5—O16	1.814 (9)	N3—C6	1.29 (3)
V5—O5	1.829 (6)	N3—C5	1.30 (3)
V5—O11	2.015 (9)	N3—H3D	0.9100
V5—O15	2.275 (9)	N3—H3E	0.9100
V6—O17	1.556 (10)	C5—H5A	0.9700
V6—O13	1.782 (10)	C5—H5B	0.9700
V6—O6	1.852 (6)	C5—H5C	0.9700
V6—O18	1.961 (9)	C6—H6A	0.9700
V6—O14	2.244 (8)	C6—H6B	0.9700
V7—O19	1.643 (9)	C6—H6C	0.9700
V7—O18	1.655 (8)	N4—C7^i^	1.428 (13)
V7—O7	1.897 (6)	N4—C7	1.428 (13)
V7—O15	2.039 (9)	N4—H4D	0.9100
V7—O14	2.137 (8)	N4—H4E	0.9100
V8—O20	1.551 (10)	C7—H7A	0.9700
V8—O16	1.766 (9)	C7—H7B	0.9700
V8—O8	1.858 (6)	C7—H7C	0.9700
V8—O19	2.025 (10)		
			
O1—V1—O3	103.1 (3)	O20—V8—O16	102.6 (5)
O1—V1—O6	101.3 (3)	O20—V8—O8	102.41 (19)
O3—V1—O6	94.4 (3)	O16—V8—O8	91.8 (2)
O1—V1—O4	101.1 (3)	O20—V8—O8^ii^	102.41 (19)
O3—V1—O4	93.0 (3)	O16—V8—O8^ii^	91.8 (2)
O6—V1—O4	154.0 (3)	O8—V8—O8^ii^	153.5 (4)
O1—V1—O7	99.3 (3)	O20—V8—O19	101.2 (5)
O3—V1—O7	156.8 (3)	O16—V8—O19	156.2 (4)
O6—V1—O7	87.0 (3)	O8—V8—O19	83.1 (2)
O4—V1—O7	76.7 (2)	O8^ii^—V8—O19	83.1 (2)
O1—V1—O14	175.5 (3)	O20—V8—O15	175.0 (5)
O3—V1—O14	81.3 (3)	O16—V8—O15	82.4 (4)
O6—V1—O14	79.5 (3)	O8—V8—O15	77.24 (19)
O4—V1—O14	77.1 (3)	O8^ii^—V8—O15	77.24 (19)
O7—V1—O14	76.3 (3)	O19—V8—O15	73.8 (3)
O2—V2—O8	102.0 (3)	O20—V8—V2^ii^	134.48 (5)
O2—V2—O5	102.3 (3)	O16—V8—V2^ii^	82.7 (2)
O8—V2—O5	93.6 (3)	O8—V8—V2^ii^	122.8 (2)
O2—V2—O7	100.3 (3)	O8^ii^—V8—V2^ii^	32.15 (18)
O8—V2—O7	91.6 (3)	O19—V8—V2^ii^	80.65 (18)
O5—V2—O7	155.2 (3)	O15—V8—V2^ii^	45.55 (4)
O2—V2—O4	99.5 (3)	V1—O3—V3	113.4 (3)
O8—V2—O4	157.0 (3)	V1—O4—V4	107.4 (3)
O5—V2—O4	89.7 (3)	V1—O4—V2	100.6 (3)
O7—V2—O4	76.6 (2)	V4—O4—V2	108.1 (3)
O2—V2—O15	176.0 (4)	V2—O5—V5	114.9 (3)
O8—V2—O15	81.0 (3)	V1—O6—V6	114.6 (3)
O5—V2—O15	80.1 (3)	V7—O7—V2	106.3 (3)
O7—V2—O15	76.8 (3)	V7—O7—V1	108.7 (3)
O4—V2—O15	77.2 (3)	V2—O7—V1	99.3 (3)
O9—V3—O13	104.4 (5)	V2—O8—V8	114.2 (3)
O9—V3—O3^ii^	101.1 (2)	V4—O10—V3	108.5 (5)
O13—V3—O3^ii^	92.5 (2)	V4—O11—V5	113.3 (5)
O9—V3—O3	101.1 (2)	V3—O13—V6	113.2 (5)
O13—V3—O3	92.5 (2)	V4—O14—V7	101.6 (3)
O3^ii^—V3—O3	155.3 (4)	V4—O14—V1	92.0 (2)
O9—V3—O10	99.1 (5)	V7—O14—V1	93.49 (19)
O13—V3—O10	156.5 (4)	V4—O14—V1^ii^	92.0 (2)
O3^ii^—V3—O10	82.9 (2)	V7—O14—V1^ii^	93.49 (19)
O3—V3—O10	82.9 (2)	V1—O14—V1^ii^	171.2 (4)
O9—V3—O14	173.5 (5)	V4—O14—V6	171.2 (4)
O13—V3—O14	82.1 (4)	V7—O14—V6	87.2 (3)
O3^ii^—V3—O14	78.4 (2)	V1—O14—V6	87.5 (2)
O3—V3—O14	78.4 (2)	V1^ii^—O14—V6	87.5 (2)
O10—V3—O14	74.4 (3)	V4—O14—V3	88.7 (3)
O9—V3—V1^ii^	133.66 (5)	V7—O14—V3	169.8 (4)
O13—V3—V1^ii^	83.1 (2)	V1—O14—V3	86.08 (19)
O3^ii^—V3—V1^ii^	32.60 (19)	V1^ii^—O14—V3	86.08 (19)
O3—V3—V1^ii^	124.5 (2)	V6—O14—V3	82.5 (3)
O10—V3—V1^ii^	80.70 (18)	V7—O15—V4	102.0 (4)
O14—V3—V1^ii^	46.22 (4)	V7—O15—V2^ii^	92.6 (2)
O11—V4—O10	108.7 (5)	V4—O15—V2^ii^	92.9 (2)
O11—V4—O4	96.8 (2)	V7—O15—V2	92.6 (2)
O10—V4—O4	98.5 (2)	V4—O15—V2	92.9 (2)
O11—V4—O4^ii^	96.8 (2)	V2^ii^—O15—V2	171.3 (5)
O10—V4—O4^ii^	98.5 (2)	V7—O15—V8	88.1 (3)
O4—V4—O4^ii^	153.5 (4)	V4—O15—V8	169.8 (4)
O11—V4—O14	162.8 (4)	V2^ii^—O15—V8	86.6 (2)
O10—V4—O14	88.5 (4)	V2—O15—V8	86.6 (2)
O4—V4—O14	80.13 (19)	V7—O15—V5	170.7 (4)
O4^ii^—V4—O14	80.13 (19)	V4—O15—V5	87.3 (3)
O11—V4—O15	85.2 (4)	V2^ii^—O15—V5	86.8 (2)
O10—V4—O15	166.1 (4)	V2—O15—V5	86.8 (2)
O4—V4—O15	79.34 (19)	V8—O15—V5	82.5 (3)
O4^ii^—V4—O15	79.34 (19)	V8—O16—V5	113.7 (5)
O14—V4—O15	77.7 (3)	V7—O18—V6	113.2 (5)
O11—V4—V1^ii^	133.00 (8)	V7—O19—V8	109.4 (5)
O10—V4—V1^ii^	86.1 (2)	H21A—O21—H21B	108.0
O4—V4—V1^ii^	125.6 (2)	C2—N1—C1	112.5 (9)
O4^ii^—V4—V1^ii^	36.19 (19)	C2—N1—H1A	109.1
O14—V4—V1^ii^	45.69 (4)	C1—N1—H1A	109.1
O15—V4—V1^ii^	84.21 (17)	C2—N1—H1B	109.1
O12—V5—O16	105.5 (5)	C1—N1—H1B	109.1
O12—V5—O5^ii^	102.2 (2)	H1A—N1—H1B	107.8
O16—V5—O5^ii^	91.1 (2)	N1—C1—H1C	109.5
O12—V5—O5	102.2 (2)	N1—C1—H1D	109.5
O16—V5—O5	91.1 (2)	H1C—C1—H1D	109.5
O5^ii^—V5—O5	153.9 (4)	N1—C1—H1E	109.5
O12—V5—O11	99.0 (5)	H1C—C1—H1E	109.5
O16—V5—O11	155.5 (4)	H1D—C1—H1E	109.5
O5^ii^—V5—O11	83.6 (2)	N1—C2—H2A	109.5
O5—V5—O11	83.6 (2)	N1—C2—H2B	109.5
O12—V5—O15	173.2 (5)	H2A—C2—H2B	109.5
O16—V5—O15	81.3 (3)	N1—C2—H2C	109.5
O5^ii^—V5—O15	77.3 (2)	H2A—C2—H2C	109.5
O5—V5—O15	77.3 (2)	H2B—C2—H2C	109.5
O11—V5—O15	74.2 (3)	C4—N2—C3	114.2 (10)
O12—V5—V2^ii^	134.56 (5)	C4—N2—H2D	108.7
O16—V5—V2^ii^	81.8 (2)	C3—N2—H2D	108.7
O5^ii^—V5—V2^ii^	32.34 (19)	C4—N2—H2E	108.7
O5—V5—V2^ii^	122.7 (2)	C3—N2—H2E	108.7
O11—V5—V2^ii^	81.0 (2)	H2D—N2—H2E	107.6
O15—V5—V2^ii^	45.40 (4)	N2—C3—H3A	109.5
O17—V6—O13	102.8 (5)	N2—C3—H3B	109.5
O17—V6—O6^ii^	102.4 (2)	H3A—C3—H3B	109.5
O13—V6—O6^ii^	91.4 (2)	N2—C3—H3C	109.5
O17—V6—O6	102.4 (2)	H3A—C3—H3C	109.5
O13—V6—O6	91.4 (2)	H3B—C3—H3C	109.5
O6^ii^—V6—O6	153.8 (4)	N2—C4—H4A	109.5
O17—V6—O18	99.9 (5)	N2—C4—H4B	109.5
O13—V6—O18	157.3 (4)	H4A—C4—H4B	109.5
O6^ii^—V6—O18	83.7 (2)	N2—C4—H4C	109.5
O6—V6—O18	83.7 (2)	H4A—C4—H4C	109.5
O17—V6—O14	175.1 (5)	H4B—C4—H4C	109.5
O13—V6—O14	82.1 (4)	C6—N3—C5	138 (3)
O6^ii^—V6—O14	77.30 (19)	C6—N3—H3D	102.6
O6—V6—O14	77.30 (19)	C5—N3—H3D	102.5
O18—V6—O14	75.1 (3)	C6—N3—H3E	102.6
O17—V6—V1^ii^	134.43 (4)	C5—N3—H3E	102.5
O13—V6—V1^ii^	81.9 (2)	H3D—N3—H3E	105.0
O6^ii^—V6—V1^ii^	32.18 (19)	N3—C5—H5A	109.5
O6—V6—V1^ii^	123.0 (2)	N3—C5—H5B	109.8
O18—V6—V1^ii^	82.20 (18)	H5A—C5—H5B	109.5
O14—V6—V1^ii^	45.67 (4)	N3—C5—H5C	109.1
O19—V7—O18	108.1 (5)	H5A—C5—H5C	109.5
O19—V7—O7^ii^	98.49 (19)	H5B—C5—H5C	109.5
O18—V7—O7^ii^	95.91 (19)	N3—C6—H6A	109.6
O19—V7—O7	98.49 (19)	N3—C6—H6B	109.6
O18—V7—O7	95.91 (19)	H6A—C6—H6B	109.5
O7^ii^—V7—O7	155.1 (4)	N3—C6—H6C	109.3
O19—V7—O15	88.6 (4)	H6A—C6—H6C	108.6
O18—V7—O15	163.2 (4)	H6B—C6—H6C	110.3
O7^ii^—V7—O15	81.19 (18)	C7^i^—N4—C7	114.6 (14)
O7—V7—O15	81.19 (18)	C7^i^—N4—H4D	108.6
O19—V7—O14	167.4 (4)	C7—N4—H4D	108.6
O18—V7—O14	84.5 (4)	C7^i^—N4—H4E	108.6
O7^ii^—V7—O14	79.73 (18)	C7—N4—H4E	108.6
O7—V7—O14	79.73 (18)	H4D—N4—H4E	107.6
O15—V7—O14	78.8 (3)	N4—C7—H7A	109.5
O19—V7—V2^ii^	86.5 (2)	N4—C7—H7B	109.5
O18—V7—V2^ii^	133.03 (8)	H7A—C7—H7B	109.5
O7^ii^—V7—V2^ii^	37.15 (17)	N4—C7—H7C	109.5
O7—V7—V2^ii^	126.58 (19)	H7A—C7—H7C	109.5
O15—V7—V2^ii^	45.60 (4)	H7B—C7—H7C	109.5
O14—V7—V2^ii^	84.63 (16)		
			
O1—V1—O3—V3	−172.9 (4)	O3—V3—O13—V6	−77.9 (2)
O6—V1—O3—V3	−70.1 (4)	O11—V4—O14—V1	94.0 (2)
O4—V1—O3—V3	85.0 (4)	O10—V4—O14—V1	−86.0 (2)
O7—V1—O3—V3	22.6 (9)	O4—V4—O14—V1	12.8 (2)
O14—V1—O3—V3	8.6 (4)	O15—V4—O14—V1	94.0 (2)
O9—V3—O3—V1	178.2 (5)	O11—V4—O14—V1^ii^	−94.0 (2)
O13—V3—O3—V1	73.0 (4)	O10—V4—O14—V1^ii^	86.0 (2)
O3^ii^—V3—O3—V1	−28.6 (13)	O4—V4—O14—V1^ii^	−175.1 (3)
O10—V3—O3—V1	−83.9 (4)	O15—V4—O14—V1^ii^	−94.0 (2)
O14—V3—O3—V1	−8.4 (4)	O19—V7—O14—V1	−92.7 (2)
V1^ii^—V3—O3—V1	−10.4 (5)	O18—V7—O14—V1	87.3 (2)
O1—V1—O4—V4	−169.4 (3)	O7^ii^—V7—O14—V1	−175.7 (3)
O3—V1—O4—V4	−65.5 (3)	O7—V7—O14—V1	−9.8 (3)
O6—V1—O4—V4	41.0 (7)	O15—V7—O14—V1	−92.7 (2)
O7—V1—O4—V4	93.5 (3)	O19—V7—O14—V1^ii^	92.7 (2)
O14—V1—O4—V4	14.8 (3)	O18—V7—O14—V1^ii^	−87.3 (2)
O1—V1—O4—V2	77.6 (3)	O7^ii^—V7—O14—V1^ii^	9.8 (3)
O3—V1—O4—V2	−178.5 (3)	O7—V7—O14—V1^ii^	175.7 (3)
O6—V1—O4—V2	−72.1 (7)	O15—V7—O14—V1^ii^	92.7 (2)
O7—V1—O4—V2	−19.5 (2)	O3—V1—O14—V4	82.1 (3)
O14—V1—O4—V2	−98.2 (3)	O6—V1—O14—V4	178.3 (3)
O11—V4—O4—V1	−178.5 (4)	O4—V1—O14—V4	−13.0 (3)
O10—V4—O4—V1	71.3 (4)	O7—V1—O14—V4	−92.3 (3)
O4^ii^—V4—O4—V1	−57.9 (9)	O3—V1—O14—V7	−176.2 (3)
O14—V4—O4—V1	−15.6 (3)	O6—V1—O14—V7	−80.0 (3)
O15—V4—O4—V1	−94.8 (3)	O4—V1—O14—V7	88.7 (3)
V1^ii^—V4—O4—V1	−19.9 (4)	O7—V1—O14—V7	9.4 (3)
O11—V4—O4—V2	−70.7 (4)	O6—V1—O14—V6	7.0 (3)
O10—V4—O4—V2	179.2 (4)	O4—V1—O14—V6	175.7 (3)
O4^ii^—V4—O4—V2	49.9 (10)	O7—V1—O14—V6	96.5 (3)
O14—V4—O4—V2	92.2 (3)	O3—V1—O14—V3	−6.5 (3)
O15—V4—O4—V2	13.1 (3)	O6—V1—O14—V3	89.7 (3)
V1^ii^—V4—O4—V2	87.9 (3)	O4—V1—O14—V3	−101.6 (3)
O2—V2—O4—V1	−78.2 (3)	O7—V1—O14—V3	179.2 (3)
O8—V2—O4—V1	80.9 (7)	O6—V6—O14—V7	86.7 (2)
O5—V2—O4—V1	179.4 (3)	O6—V6—O14—V1	−6.9 (2)
O7—V2—O4—V1	20.2 (2)	O6^ii^—V6—O14—V1^ii^	6.9 (2)
O15—V2—O4—V1	99.5 (3)	O6—V6—O14—V1^ii^	−179.6 (3)
O2—V2—O4—V4	169.3 (3)	O3—V3—O14—V4	−85.8 (2)
O8—V2—O4—V4	−31.6 (9)	O3—V3—O14—V7	94.2 (2)
O5—V2—O4—V4	66.9 (3)	O13—V3—O14—V1	−87.9 (2)
O7—V2—O4—V4	−92.3 (3)	O3^ii^—V3—O14—V1	177.8 (3)
O15—V2—O4—V4	−13.0 (3)	O3—V3—O14—V1	6.3 (3)
O2—V2—O5—V5	174.9 (4)	O10—V3—O14—V1	92.1 (2)
O8—V2—O5—V5	71.8 (4)	O13—V3—O14—V1^ii^	87.9 (2)
O7—V2—O5—V5	−30.0 (8)	O3^ii^—V3—O14—V1^ii^	−6.3 (3)
O4—V2—O5—V5	−85.5 (3)	O3—V3—O14—V1^ii^	−177.8 (3)
O15—V2—O5—V5	−8.4 (4)	O10—V3—O14—V1^ii^	−92.1 (2)
O12—V5—O5—V2	−178.8 (5)	O3—V3—O14—V6	94.2 (2)
O16—V5—O5—V2	−72.7 (4)	O7—V7—O15—V2^ii^	−174.7 (3)
O5^ii^—V5—O5—V2	22.2 (11)	O14—V7—O15—V2^ii^	−93.5 (2)
O11—V5—O5—V2	83.3 (4)	O19—V7—O15—V2	−86.5 (2)
O15—V5—O5—V2	8.2 (3)	O18—V7—O15—V2	93.5 (2)
V2^ii^—V5—O5—V2	8.3 (4)	O7^ii^—V7—O15—V2	174.7 (3)
O1—V1—O6—V6	175.2 (4)	O7—V7—O15—V2	12.3 (3)
O3—V1—O6—V6	70.9 (4)	O14—V7—O15—V2	93.5 (2)
O4—V1—O6—V6	−35.3 (8)	O4—V4—O15—V7	82.1 (2)
O7—V1—O6—V6	−85.9 (3)	O14—V4—O15—V7	0.0
O14—V1—O6—V6	−9.4 (3)	O11—V4—O15—V2^ii^	−86.7 (2)
O17—V6—O6—V1	−175.8 (5)	O10—V4—O15—V2^ii^	93.3 (2)
O13—V6—O6—V1	−72.4 (4)	O4—V4—O15—V2^ii^	175.4 (3)
O6^ii^—V6—O6—V1	23.8 (12)	O4^ii^—V4—O15—V2^ii^	11.2 (3)
O18—V6—O6—V1	85.4 (4)	O14—V4—O15—V2^ii^	93.3 (2)
O14—V6—O6—V1	9.3 (3)	V1^ii^—V4—O15—V2^ii^	47.5 (2)
O19—V7—O7—V2	72.7 (4)	O11—V4—O15—V2	86.7 (2)
O18—V7—O7—V2	−177.9 (4)	O10—V4—O15—V2	−93.3 (2)
O7^ii^—V7—O7—V2	−59.9 (9)	O4—V4—O15—V2	−11.2 (3)
O15—V7—O7—V2	−14.6 (3)	O4^ii^—V4—O15—V2	−175.4 (3)
O14—V7—O7—V2	−94.7 (3)	O14—V4—O15—V2	−93.3 (2)
O19—V7—O7—V1	178.7 (4)	O4—V4—O15—V8	−97.9 (2)
O18—V7—O7—V1	−71.9 (4)	O4—V4—O15—V5	−97.9 (2)
O7^ii^—V7—O7—V1	46.1 (10)	O8—V2—O15—V7	81.5 (3)
O15—V7—O7—V1	91.5 (3)	O5—V2—O15—V7	176.8 (3)
O14—V7—O7—V1	11.4 (3)	O7—V2—O15—V7	−12.3 (3)
O2—V2—O7—V7	−169.1 (3)	O4—V2—O15—V7	−91.3 (3)
O8—V2—O7—V7	−66.6 (3)	O8—V2—O15—V4	−176.3 (3)
O5—V2—O7—V7	35.6 (7)	O5—V2—O15—V4	−81.0 (3)
O4—V2—O7—V7	93.5 (3)	O7—V2—O15—V4	89.9 (3)
O15—V2—O7—V7	13.8 (3)	O4—V2—O15—V4	10.9 (3)
O2—V2—O7—V1	78.2 (3)	O8—V2—O15—V8	−6.5 (3)
O8—V2—O7—V1	−179.3 (3)	O5—V2—O15—V8	88.8 (3)
O5—V2—O7—V1	−77.1 (7)	O7—V2—O15—V8	−100.3 (3)
O4—V2—O7—V1	−19.2 (2)	O4—V2—O15—V8	−179.2 (3)
O15—V2—O7—V1	−98.9 (3)	O8—V2—O15—V5	−89.2 (3)
O1—V1—O7—V7	169.8 (3)	O5—V2—O15—V5	6.2 (3)
O3—V1—O7—V7	−25.5 (9)	O7—V2—O15—V5	177.0 (3)
O6—V1—O7—V7	68.7 (3)	O4—V2—O15—V5	98.1 (3)
O4—V1—O7—V7	−90.9 (3)	O8—V8—O15—V7	−86.5 (2)
O14—V1—O7—V7	−11.2 (3)	O8^ii^—V8—O15—V7	86.5 (2)
O1—V1—O7—V2	−79.4 (3)	O8—V8—O15—V4	93.5 (2)
O3—V1—O7—V2	85.3 (7)	O8^ii^—V8—O15—V4	−93.5 (2)
O6—V1—O7—V2	179.6 (3)	O16—V8—O15—V2^ii^	87.2 (2)
O4—V1—O7—V2	19.9 (2)	O8—V8—O15—V2^ii^	−179.2 (3)
O14—V1—O7—V2	99.6 (3)	O8^ii^—V8—O15—V2^ii^	−6.3 (3)
O2—V2—O8—V8	−174.0 (4)	O19—V8—O15—V2^ii^	−92.8 (2)
O5—V2—O8—V8	−70.6 (4)	O16—V8—O15—V2	−87.2 (2)
O7—V2—O8—V8	85.1 (4)	O8—V8—O15—V2	6.3 (3)
O4—V2—O8—V8	27.1 (9)	O8^ii^—V8—O15—V2	179.2 (3)
O15—V2—O8—V8	8.7 (4)	O19—V8—O15—V2	92.8 (2)
O20—V8—O8—V2	176.6 (5)	O8—V8—O15—V5	93.5 (2)
O16—V8—O8—V2	73.4 (4)	O5—V5—O15—V4	86.9 (2)
O8^ii^—V8—O8—V2	−24.1 (12)	O16—V5—O15—V2^ii^	−87.0 (2)
O19—V8—O8—V2	−83.3 (4)	O5—V5—O15—V2^ii^	179.9 (3)
O15—V8—O8—V2	−8.5 (3)	O16—V5—O15—V2	87.0 (2)
O4—V4—O10—V3	−79.8 (2)	O5—V5—O15—V2	−6.1 (3)
O3^ii^—V3—O10—V4	−79.9 (2)	O11—V5—O15—V2	−93.0 (2)
O3—V3—O10—V4	79.9 (2)	O5—V5—O15—V8	−93.1 (2)
O5—V5—O11—V4	−78.6 (2)	O6—V6—O18—V7	−78.5 (2)
O15—V5—O11—V4	0.0	V1^ii^—V6—O18—V7	46.10 (4)

Symmetry codes: (i) −*x*+3/2, *y*, −*z*+3/2; (ii) −*x*+1, *y*, *z*.

### Hydrogen-bond geometry (Å, °)

**Table:**

*D*—H···*A*	*D*—H	H···*A*	*D*···*A*	*D*—H···*A*
O21—H21B···O10^iii^	0.85	2.30	2.950 (19)	133
O21—H21A···O16	0.85	2.26	3.101 (18)	171
N4—H4D···O5^i^	0.91	1.77	2.673 (8)	170
N4—H4E···O5	0.91	1.77	2.673 (8)	170
N2—H2D···O6	0.91	1.88	2.759 (10)	162
N2—H2E···O6^iv^	0.91	2.22	2.986 (10)	141
N2—H2E···O7^iv^	0.91	2.05	2.812 (9)	140
N1—H1A···O8^v^	0.91	1.86	2.732 (10)	161
N1—H1B···O3	0.91	1.79	2.674 (10)	164

Symmetry codes: (iii) *x*, *y*−1/2, −*z*+3/2; (i) −*x*+3/2, *y*, −*z*+3/2; (iv) −*x*+3/2, −*y*+3/2, −*z*+2; (v) −*x*+3/2, *y*+1/2, *z*.
